# High-Knee-Flexion Posture Recognition Using Multi-Dimensional Dynamic Time Warping on Inertial Sensor Data

**DOI:** 10.3390/s25041083

**Published:** 2025-02-11

**Authors:** Annemarie F. Laudanski, Arne Küderle, Felix Kluge, Bjoern M. Eskofier, Stacey M. Acker

**Affiliations:** 1 Biomechanics of Human Mobility Laboratory, Department of Kinesiology and Health Sciences, University of Waterloo, Waterloo, ON N2L 3G1, Canada; stacey.acker@uwaterloo.ca; 2 Machine Learning and Data Analytics Laboratory, Department of Artificial Intelligence in Biomedical Engineering, Friedrich-Alexander Universität, Erlangen-Nürnberg (FA), 91054 Erlangen, Bavaria, Germany; arne.kuederle@fau.de (A.K.); bjoern.eskofier@fau.de (B.M.E.)

**Keywords:** inertial sensors, posture classification, accelerometer, gyroscope, dynamic time warping, knee osteoarthritis, occupational ergonomics, high-knee flexion

## Abstract

Relating continuously collected inertial data to the activities or postures performed by the sensor wearer requires pattern recognition or machine-learning-based algorithms, accounting for the temporal and scale variability present in human movements. The objective of this study was to develop a sensor-based framework for the detection and measurement of high-flexion postures frequently adopted in occupational settings. IMU-based joint angle estimates for the ankle, knee, and hip were time and scale normalized prior to being input to a multi-dimensional Dynamic Time Warping (mDTW) distance-based Nearest Neighbour algorithm for the identification of twelve postures. Data from 50 participants were divided to develop and evaluate the mDTW model. Overall accuracies of 82.3% and 55.6% were reached when classifying movements from the testing and validation datasets, respectively, which increased to 86% and 74.6% when adjusting for imbalances between classification groups. The highest misclassification rates occurred between flatfoot squatting, heels-up squatting, and stooping, while the model was incapable of identifying sequences of walking based on a single stride template. The developed mDTW model proved robust in identifying high-flexion postures performed by participants both included and precluded from algorithm development, indicating its strong potential for the quantitative measurement of postural adoption in real-world settings.

## 1. Introduction

Knee osteoarthritis (OA) is a debilitating disorder of increasing prevalence, with repetitive cyclic or prolonged joint loading having been associated with the initiation and progression of joint tissue degradation leading to OA [1,2,3]. These loading patterns are often observed during high-knee-flexion activities (such as kneeling and squatting), where the knee flexion angle exceeds 120°. Despite their association with increased risk of musculoskeletal injuries [4,5,6,7], workers across many occupations are often required to perform repetitive and sustained high-knee-flexion motions for a significant portion of their working hours. These repetitive and prolonged occupational exposures may contribute to altering the biomechanical and neuromuscular balance within the joint and, over time, may lead to work restrictions, work–time loss, or even work leave [8]. In fact, knee OA in North America has been shown to be the most common reason for total knee replacement, and the rapidly growing demand for these surgeries stemming from cases of OA is likely to present a growing problem for public health systems in the years to come [9].

Despite the growing incidence of knee OA worldwide, until recently no methods have been available for the objective evaluation of high-knee-flexion exposures in occupational settings associated with acute and chronic injuries potentially related to knee OA development, given that most monitoring or motion capture systems require line of sight visibility of the participant, which is often not possible in the workplace. Wearable sensors, often composed of accelerometers and gyroscopes, now offer a minimally intrusive means of measuring occupational kinematics over multiple workdays [10,11,12,13]; however, the interpretation of their data as they relate to occupational exposures is non-trivial. These sensors provide only information regarding the kinematics of specific body segments, however, to associate those kinematics with adopted postures requires sophisticated postural recognition software. Therefore, recent studies have sought to apply pattern recognition and machine learning algorithms to interpret the raw sensor data for activity identification and monitoring [14,15,16,17,18].

Activity classification generally involves a two-stage process wherein representative features are extracted from sensor data and subsequently input to a classification algorithm to associate these data with a specific movement or activity [19]. The goal in deriving classification features from sensor-based signals is to reduce the dimensionality of the signal while ensuring maximum class separability, through feature complexity and robustness to variations over time [20]. K Nearest Neighbour (*k*NN) classification algorithms have been used in many previous activity classification studies based on wearable sensor signals [19,21,22,23] due to their computational simplicity by comparing the distance of an unknown point to labelled datapoints (often referred to as model building or training datapoints) each corresponding to a specific motion.

Direct classification of time-series data has recently become of interest [12,24,25,26,27], wherein the distance between two samples (now time-series curves) are computed as input for classification. One challenge in classifying time series data however is the effect of temporal variations in movements on classification accuracy. By employing non-linear corrections between signals in a process referred to as alignment, these variations can be mitigated prior to calculating the distance between samples, or alternatively, the distance between samples can serve as an alternative distance metric by which the classification can be performed [24,27].

For classification, alignment-based distance metrics, such as Dynamic Time Warping (DTW) distances, are typically calculated between some unknown signal and a previously labelled signal template [28]. Template-based DTW classifiers have utilized either raw accelerometer or gyroscope data in applications of stride segmentation within free walking bouts in both healthy and clinical populations and in the recognition of hand gestures during activities of daily living [25,29,30]. Multi-dimensional Dynamic Time Warping (mDTW), an extension of the traditional DTW approach, allows for the combination of DTW-based distances across multiple inputs (e.g., across multiple sensor axes) to increase the separability of samples, thereby increasing the calibration accuracy [24,31]. While these approaches have been used for the identification of sitting, standing, lying down, level walking, and stair climbing [30,32,33], no studies to date have applied DTW techniques to the identification of high-knee-flexion postures.

Therefore, the objective of this study was to develop a sensor-based framework for the detection and measurement of high-flexion postures frequently adopted in occupational settings. To accomplish this, we proposed the use of a mDTW-distance-based *k*NN classification algorithm that might ultimately be used for the continuous identification of high-flexion exposures in occupational settings.

## 2. Materials and Methods

### 2.1. Participants and Experimental Protocol

Fifty participants (18 males/32 females, height: 1.11 ± 0.83 m, mass: 73.70 ± 17.78 kg, age: 21.14 ± 3.76 years) were recruited for this study approved by the University of Waterloo Research Ethics Board, and informed consent was obtained prior to each collection [34]. Participation was limited to those who self-identified as capable of kneeling or squatting without difficulty or pain; however, participants were not required to have any prior history of interacting with or caring for children in order to participate. Study participants were primarily young adults enrolled in a kinesiology undergraduate or graduate program, and as such, the majority had received prior training on ergonomic practices associated with postural adoption in workplace settings. On a 3-point scale of daily squatting regularity, where scores from 1 to 3 equated to rare, occasional, or regular daily adoption of squatting postures, participants reported a mean score of 1.96 ± 0.67.

**Figure 1:**
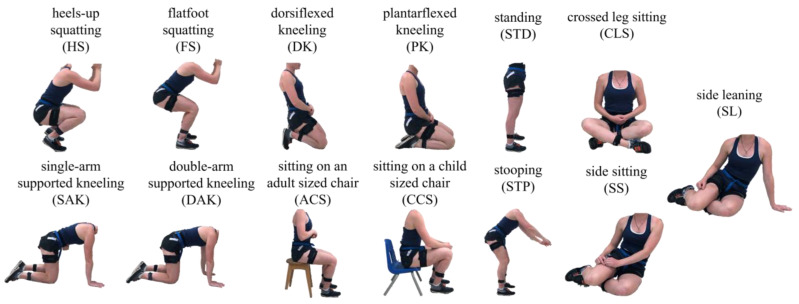
Depiction of the 13 occupational-inspired motions performed in this study in their fully flexed position. Note that SS and SL are displayed to the left, however were additionally performed to the participant’s right.

Participants were instrumented with seven wireless IMUs (Xsens MTw Awinda, Movella Inc., Henderson, NV, USA, Gyro: 2000 deg/s, Acc: 16 G, sampling frequency: 60 Hz) attached bilaterally to the superior aspect of the feet, the lateral aspect of the shanks and thighs, as well as over the base of the sacrum. Each participant completed a 5 s standing trial in addition to functional hip [35] and functional knee (isolated cyclic flexion/extension motion of the lower leg while the upper leg was held parallel to the ground) motion trials. Participants then completed three 10 m walking trials at a self-selected pace and performed three repetitions of 13 randomized movements commonly adopted in occupational settings (Table 1, Figure 1). These movements included heels-up squatting (HS), flatfoot squatting (FS), dorsiflexed kneeling (DK), plantarflexed kneeling (PK), single arm supported kneeling (SAK), double arm supported kneeling (DAK), sitting on an adult-sized chair (ACS), sitting on a child-sized chair (CCS), crossed leg sitting (CLS), side sitting (SS), side leaning (SL), stooping (STP), and standing (STD). Each motion trial, save the seated trials, consisted of participants taking a step forward, leading with their dominant leg, before transitioning into the movement from standing, holding the fully flexed pose for 5 s, transitioning back to standing, and taking a step backwards again leading with their dominant leg. For the seated motions, participants began from standing, transitioned into the movement, held the fully flexed pose for 5 s, and transitioned back to standing. All measurements were taken in a controlled laboratory environment and prior to beginning the study, participants were introduced to each movement with a picture and their descriptions (Table 1) and given the opportunity to practice until they felt comfortable. In the event that during the collection participants could not remember the posture requested, they were read the same description and allowed to practice should they request it.

### 2.2. Sensor Signal Processing and Data Labelling

Linear acceleration and angular velocity data were used to calculate flexion–extension angles for the ankle, knee, and hip [36,37,38]. Each IMU applied an onboard Strap-Down Integration algorithm to the raw sensor data to decrease the sampling rates from 1000 Hz to 60 Hz while maintaining high fidelity in the recorded signals following the application of a low-pass filter at a bandwidth of 184 Hz. No additional sensor fusion or filtering was applied to these signals prior to the processing herein described. The start and end points of each motion were identified manually based on the right knee flexion–extension angles. For high-flexion motions, each trial started and ended with a static standing period, so the start and end of each motion was visually identified as closely as possible to the last or first static frame of standing, respectively, noting that the visual identification may have resulted in the inclusion of one or two frames of additional static data in this identification. Each walking trial was divided into three gait cycles, with each cycle—as closely as possible—bounded by the two subsequent instances of maximum knee extension (which correspond to the occurrence of heel strike).

Each trial was labelled according to the motion performed. DAK and SAK trials were combined for classification and simply labelled as supported kneeling (SK), while all SS and SL motions were labelled as side sitting (SS) given the similarity in lower limb joint kinematics observed between movements. As such, the classification model was designed for the identification of 12 independent motion classes.

### 2.3. mDTW Distance Metric Development for kNN Classification

To classify the movement sequences of interest in this study, a K Nearest Neighbour classification algorithm based on mDTW distance metrics was developed. DTW distances were calculated between a series of labelled movement class templates and the unknown movement sequences for each joint from each leg, and subsequently combined into one mDTW distance metric as input to the *k*NN classifier. The workflow utilized for the development of these distance metrics will be described in the following section.

**Figure 2:**
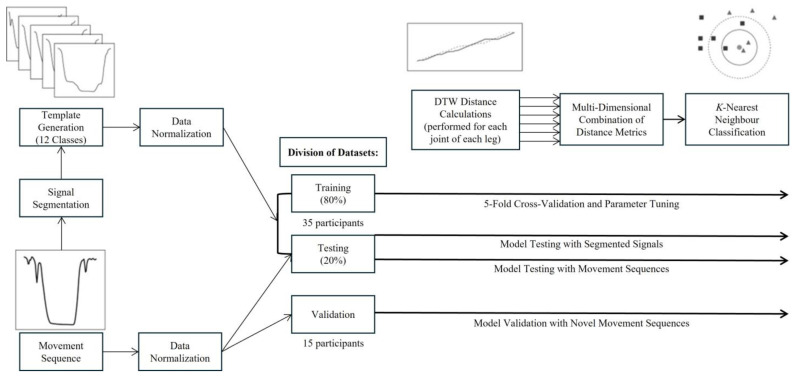
Signal processing workflow for the mDTW *k*NN algorithm. The DTW distances for each joint were calculated between a single normalized trial (referred to as a movement sequence) and the normalized templates for the 12 movement classes included in the developed model. A smaller distance between the sequence and template would represent greater similarity between waveforms. Once each movement sequence had been compared to all templates for the corresponding joint and leg, the DTW distances were combined across joints using custom weighting factors such that the movement class would ultimately be determined based on the mDTW distance.

The workflow for this proposed mDTW *k*NN algorithm has been presented in Figure 2. Movement trials from a subset of 35 randomly selected participants were combined to create the algorithm building dataset (for training and testing), while the remaining 15 participants’ data were withheld for model validation.

A series of movement sequences *S_trial_* were created such that each contained data from a single movement trial, from which the occupational-inspired motion would be identified, as in Equation (1). Given that each motion was collected in isolation, each *S_trial_* represents only one motion type, and contains time-series signals representing the left and right ankle, knee, and hip flexion–extension angle data, each of length *N*.

(1) $$S_{trial}=\left(s_{0}\ldots s_{N-1}\right)=\left(\begin{array}{cccc} s_{RKnee,0} & s_{RKnee,1} & \cdots & s_{RKnee,N-1}\\ s_{LKnee,0} & s_{LKnee,1} & \cdots & s_{LKnee,N-1}\\ s_{RAnkle,0} & s_{RAnkle,1} & \cdots & s_{RAnkle,N-1}\\ \vdots & \vdots & \ddots & \vdots \\ s_{LHip,0} & s_{LHip,1} & \cdots & s_{LHip,N-1} \end{array}\right)$$

**Figure 3:**
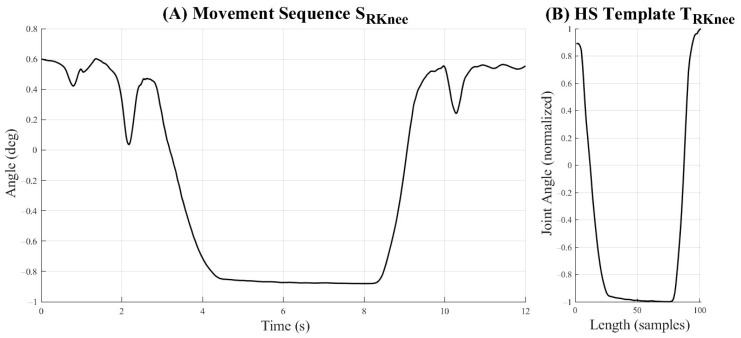
A representative unnormalized movement sequence *S_RKnee_* for the flexion angle of the right knee during a heels-up squatting (HS) motion trial (**A**) along with the corresponding heels-up squat template *T_RKnee_* generated from this sequence for the right knee (**B**).

**Figure 4:**
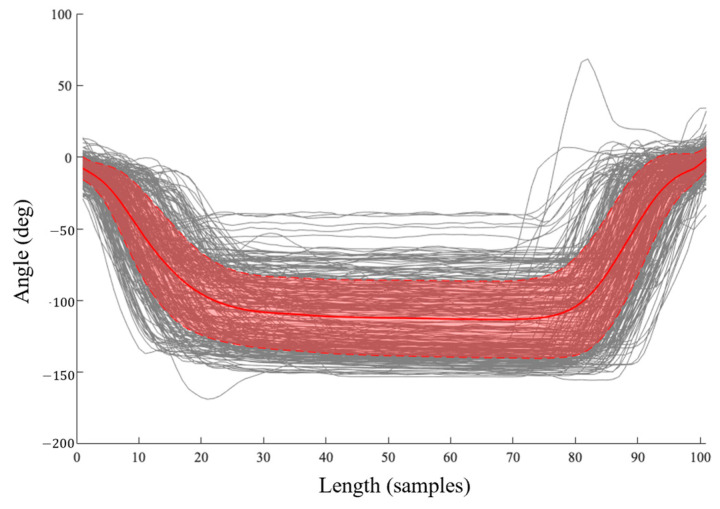
Templates generated for heels-up squatting based on the right knee across all trials and participants, with mean and standard deviation curves overlayed in red.

A series of templates T could then be generated from the 35 participants’ motion data through the manual segmentation to populate the *k*NN labelled feature space. The dataset here analyzed provided 1911 templates across the 11 occupational-inspired movements and gait trials, with each template consisting of the left and right ankle, knee, and hip flexion–extension angle data. All segmented trials were subsequently linearly interpolated to a length of 101 samples for each joint angle. All movements waveforms not used for templating were also normalized through linear interpolation to a length of 101 samples; however, no segmentation was performed on these data such that they might be more representative of angular profiles from movement performance in real-world settings rather than in isolation. All segmented and unsegmented data were scale normalized to a range of [−1, 1]. A representative movement sequence and template based on the right knee angle during HS can be seen in Figure 3. We here note that a large degree of variability existed in the joint angle data collected across all participants, as visually demonstrated for a representative movement class in Figure 4. This variability within templates included in the labelled feature space is the result of differences in the speed of ascent and descent of participants as well as the depth of knee flexion angles achieved by each within a single movement for this study’s population.

For each comparison between a given movement sequence *S* and a template *T* based on data from a single joint, a measure of distance, *D*, was calculated based on similarities between the waveforms [39,40,41]. *D* is therefore calculated as a *M* × *N* matrix, where *M* is the length of the template *T* (representing the rows), and *N* is the length of the movement sequence *S* (representing the columns). Given that both the templates and the sequences were normalized to 101 points, each *D* is therefore calculated as a 101 × 101 matrix. Each element of *D* is calculated as the distance between each combination of elements from *T* and *S* using the Euclidean norm such that:

(2) $$D\left(m,n\right)=\sqrt{{\left(t_{m}-s_{n}\right)}^{2}}\forall m\in \left\{1,\ldots ,M\right\},n\in \left\{1,\ldots ,N\right\},$$

$$D=\left[\begin{array}{cccccc} d_{11} & d_{21} & & \ldots & & d_{M1}\\ d_{12} & & & & & \\ \vdots & & & d_{mn} & & \vdots \\ d_{1N} & & & \ldots & & d_{MN} \end{array}\right].$$

When *t_m_* and *s_n_* are similar, the local value of *d* would be small, representing a strong similarity between measures, while large values of *d* represent less similarity between measures. The top row of *D* represents the distance between the beginning of the template *T* and the complete sequence *S* while the bottom row represents the distance between the end of the template and *S*. The ultimate DTW distance can then be calculated based on a path through the matrix *D*, which is parameterized by two isometric sequences, *ix* and *iy*, representing the warped paths of *S* and *T*, which minimize the following:

(3) $$D=\sum_{\begin{array}{c} m\in ix\\ n\in iy \end{array}}d_{mn}(MN).$$

**Figure 5:**
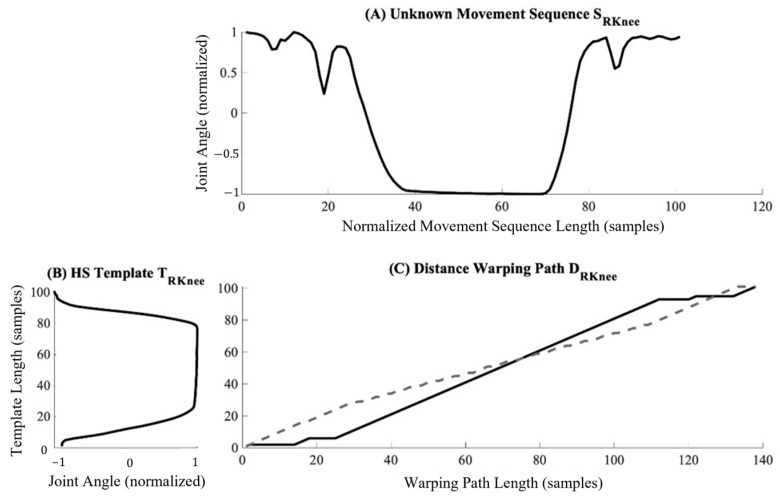
Representative warping paths *ix* and *iy* derived during the calculation of *D_RKnee_* (**C**) based on only the right knee between the movement sequence *S_RKnee_* (**A**) and the template *T_RKnee_* (**B**) for a heels-up squatting motion trial. Each signal is warped such that the highest level of similarity between waveforms can be achieved. Note that in warping the signals, the length of the final sample may be longer than either original waveform. The dotted and solid lines represent the warped recreations of the movement sequence and template, respectively.

The warped paths, *ix* and *iy*, are calculated from *d*_11_ to *d_MN_* and advance through the matrix in a series of moves described by the following constraints:

(4) $$\left(m,n\right)\to \left(m+1,n\right)\;if\;d_{m+1,n}<\left(d_{m,n+1}\;and\;d_{m+1,n+1}\right),$$

(5) $$\left(m,n\right)\to \left(m,n+1\right)\;if\;d_{m,n+1}<\left(d_{m+1,n}\;and\;d_{m+1,n+1}\right),$$

(6) $$\left(m,n\right)\to \left(m+1,n+1\right)\;if\;d_{m+1,n+1}<\left(d_{m+1,n}\;and\;d_{m,n+1}\right),$$

such that the final distance, *d_MN_*, represents the DTW distance between the waveforms *S* and *T*. For this study, warping paths were constrained to be within 50 samples of a straight line fit between waveforms, which for templates and sequences of 101 points, represents a maximum warping of 49.5%. This warping path constraint was selected as it was found to maximize classification accuracy when iterating in intervals of 10 from 0 to 100 through the training dataset. An example of the warping path calculated between a given movement sequence and template for the right knee is shown in Figure 5.

A DTW distance was computed for each of the six joints, and subsequently, a weighted sum of these distances was calculated to generate a new multi-dimensional distance metric. Weightings for each distance element were initially selected through manual iteration, wherein iterations were performed in intervals of 0.05 from 0.25 to 0.75 (motivated by the scale of the joint angles experienced by each joint and their contributions to the movements in question) while prescribing a value of 1.00 to $D_{RKnee}$, such that the initial weightings utilized—which minimized the distance metric in the training dataset—were as follows:

(7) $$D_{mDTW}=0.25\cdot D_{LAnkle}+0.25\cdot D_{RAnkle}+0.75\cdot D_{LKnee}+D_{RKnee}+0.75\cdot D_{LAHip}+0.75\cdot D_{RHip}.$$

This distance metric could be treated the same as any distance measure input to a k-Nearest Neighbour algorithm for the classification of derived features (rather than the classification of continuous time domain data as proposed herein), and thus would allow for the identification of occupational-inspired motions based on inertial estimates of lower limb flexion–extension angles.

### 2.4. kNN Classification Algorithm Training and Testing

Templates built from the randomly selected 35 participants’ data were combined with their appropriate motion labels to create the algorithm building dataset for the training and testing of a *k*NN cross-validated classifier. This dataset was relied upon for iterative parameter tuning and ultimately populated the labelled feature space from which classifications would be made. Therefore, for our twelve-activity classification problem, each motion would be identified as a different class, α, such that α_i_ = 0, 1, 2, …, 11. It is advised that each movement class should be equally represented within the algorithm building dataset so as to ensure the final parameters that the iterative algorithm building process selected were non-biased towards any class or condition [40]. Due to the grouping of similar movements, it is noted that the number of templates included in this dataset for SS and SK was significantly greater than those included for the other motions.

A holdout validation method was employed to train and test the classifier, such that 80% of the templates from each motion were randomly allocated as training data while the remaining 20% were withheld for testing [42]. The training data were then partitioned into five equal parts, or folds, such that five-fold cross-validation could be used to develop the *k*NN classifier and obtain an initial estimate of classification accuracy. Permutations of four folds were therefore used to populate the feature space of labelled datapoints, while the fifth fold was reserved for testing. In this way, the accuracy of the initial classifications through the *k*NN classifier were obtained from the average of the five permutations when using a value of *k* = 1, and the distances between samples and reference templates calculated based on Equation (7).

Following the initial classifier development, the model performance was evaluated across multiple values of *k*, logarithmically spaced between 1 and the length of the training dataset, 1911, to determine the optimal number of nearest neighbours for this classification problem. The five-fold classification errors associated with each *k* value were then plotted and the value of *k*, which minimized the cross-validated losses selected as optimal. The model was found to classify most accurately when a value of 1 was attributed to *k*.

Subsequently, the weighting parameters used in the mDTW distance metric calculation were also optimized using the cross-validated model. Each weighting coefficient was varied within ±0.045 of the initial values presented in Equation (7) with a step size of 0.015 for the left ankle, knee, and hip, and the right ankle and hip, while for the right knee the weighting coefficient was varied between 0.7 and 1. These permutations were tested simultaneously such that the combination of coefficients, which best minimized the five-fold classification losses, was selected, thereby increasing the predictivity of the model. The final mDTW distance was calculated as follows:

(8) $$D_{mDTW}=0.26\cdot D_{LAnkle}+0.20\cdot D_{RAnkle}+0.72\cdot D_{LKnee}+D_{RKnee}+0.67\cdot D_{LAHip}+0.76\cdot D_{RHip}$$

Once these parameters had been tuned, a final *k*NN model was developed using all development data and subsequently evaluated using the test samples (the 20% of templates held-back from the algorithm building dataset, which will be referred to as segmented trials), as well as using the corresponding movement sequences which had not undergone segmentation, in order to determine the model’s performance when identifying unknown waveforms—representing both movements in isolation and motion segments including additional steps—from individuals on which the classifier was trained.

Finally, movement sequences for all motions from each of the 15 participants withheld for validation were classified to evaluate the classification accuracy when the model was provided with novel data.

The classification performance of all models was quantified as the ratio of correct classifications to the total number of classifications. While the rate of successfully identifying each motion based on chance would be 7.7% (100%/the number of motions to be classified), the goal of the developed classification framework was to identify each motion as accurately as possible. To this end, sensitivity and specificity were calculated for each motion to determine whether any motions suffered from greater risk of classification errors within the development, testing, and validation models [42].

Sensitivity, a measure of the true positive classifications which can also be referred to as recall, was calculated as the proportion of waveforms of a certain motion correctly identified as follows:

(9) $$Sensitivity=\frac{TP}{TP+FN},$$

where *TP*, or true positive, are the number of sequences within a given motion correctly identified as such, and *FN*, or false negative, are the number of sequences within a given motion incorrectly identified as other motions. Therefore, the term (*TP* + *FN*) represents the total number of sequences for the given motion [42]. A high sensitivity value would represent the ability to classify a motion with few false negatives but does not consider the number of false positives.

Specificity, a measure of true negative classifications, was calculated as the proportion of feature sets not belonging to a specific motion correctly identified as other motions as follows:

(10) $$Specificity=\frac{TN}{TN+FP},$$

where *TN*, or true negative, are the number of sequences not belonging to a given motion correctly classified as belonging to the other motions, and *FP*, or false positive, are the number of sequences not belonging to a given motion which are incorrectly classified as the motion in question. Therefore, the term (*TN* + *FP*) represents the total number of sequences belonging to all other motions [42]. A high specificity value would represent the ability to classify a motion with few false positives, however, this does not consider the number of false negatives.

Given the presence of imbalanced templates across motions, balanced accuracy, ${Acc}_{Bal}$*_l_*, was also calculated as follows:

(11) $${Acc}_{Bal}=\frac{1}{2}\cdot \frac{TP}{TP+FN}+\frac{1}{2}\cdot \frac{TN}{TN+FP},$$

This metric combines both the sensitivity and specificity of a given model, and limits inflated accuracy scores by combining the number of true positives (*TP*), true negatives (*TN*), false positives (*FP*), and false negatives (*FN*) into a single representative value [43]. All classifications and analyses of error were completed using Matlab 9.13 (The Mathworks, Release R2022b, Natick, MA, USA).

## 3. Results

### 3.1. mDTW kNN Classification Algorithm 5-Fold Cross-Validated Training

Through five-fold cross-validation, the initial mDTW *k*NN algorithm was developed based on movement templates of lower limb flexion–extension angles calculated from inertial sensor data across twelve motions from 35 participants. Initial classification accuracy following cross-validation of the templates included in the algorithm building dataset was found to be 82.2%. Sensitivity, specificity, and balanced accuracy values for all classifiers are presented in Table 2A, Table 3, and Table 4, respectively. Despite imbalances in the number of templates included for each motion, consistent specificity values were reported across classes based on this model (mean specificity was found to be 98.1% ± 1.3%); however, when balancing for the sample sizes using Equation (11), the balanced accuracy of the model was found to be 89.5%. In this initial classifier, the highest levels of misclassification were observed between flatfoot and heels-up squatting (with 81.6% and 64.9% of classifications representing *TP* predictions, respectively, while *FN* classifications, misidentified as the opposite motion, were reported as 10.5% and 27.0% of the classifications, respectively).

### 3.2. Parameter Tuning and Algorithm Testing

The parameters selected for *k* and for the weightings used in the mDTW distance metric calculation (Equation (7)) were subsequently tuned to reduce the cross-validated classification errors of this initial model (results of which are presented in Equation (8)). Subsequently, a new *k*NN model was developed using the entirety of the training data templates to populate the feature space of labelled datapoints. This model was first evaluated using the segmented trials corresponding to the 20% of data withheld for testing from the algorithm building dataset, results of which are presented in Table 2B, Table 3 and Table 4. The classification accuracy of this new model increased to 84.1% overall, with a balanced accuracy of 88%. These results suggest that tuning the parameters in Equation (8) and increasing the size of the feature space of labelled datapoints for comparison resulted in increased classification sensitivity, which can be observed through the *TP* classifications for all movement classes save standing.

The tuned classifier’s performance was next evaluated using the same testing data in the form of movement sequences. The classification accuracy achieved for these data was 82.3% overall, with a mean balanced accuracy of 86% across all motions (Table 4), suggesting that the classifier could identify novel movement sequences performed by individuals for which it was trained, with only minor decreases in overall accuracy achieved when classifying these movement sequences when compared to the classification of segmented motion trials (motion templates). Sensitivity and specificity values for each motion, presented in Table 2C and Table 4, revealed that the greatest classification accuracies were achieved when identifying kneeling and floor sitting motions. The most common misclassifications occurred between flatfoot squatting, heels-up squatting, and stooping, where the squatting motions were frequently identified as stooping (where the *FN* rates of flatfoot and heels-up squatting identified as stooping were 28.9% and 26.3%, respectively), yet stooping was rarely misclassified as squatting (where the *FN* rates of stooping identified as flatfoot and heels-up squatting were 0.0% and 4.8%, respectively). It is noted however that the classification specificities were consistent across all motions (98.1% ± 2.0%), representing the classifiers’ strong ability to reject *FPs*. This classification model also revealed that while walking could be identified with 100% accuracy when classifying segmented trials representing individual steps, templates representing a single step were not sufficient for the identification of walking sequences which may include multiple steps.

### 3.3. Algorithm Validation with Novel Participant Data

Finally, the tuned and tested classification model was used to predict the occupational-inspired motions for movement sequences from fifteen novel participants (withheld from the algorithm building dataset). The classifier was found to be 55.6% accurate when identifying occupational-inspired motions from novel participants; however, when considering the balanced accuracy (Table 4), this value increased to 74.6%. Specificity values diminished when classifying novel participant waveforms (93.5% ± 3.6%, Table 3); however, the greatest changes were observed in sensitivity, as presented in Table 2D, with a mean classification sensitivity of 55.6% ± 23% reported across all 12 movement classes. When gait trials were excluded from this calculation, a mean sensitivity of 60.7% ± 15% was reported across the remaining 11 classes.

This model, however, revealed elevated levels of misclassifications between motions of similar types (i.e., between dorsiflexed, plantarflexed, and supported kneeling; flatfoot and heels-up squatting; sitting on adult or child sized chairs; and crossed leg or side sitting). By grouping these motions by similarities, overall classification accuracy was increased to 75.7%, with sensitivity, specificity, and balanced accuracy values for these groupings presented in Table 5. It is likely that the decreased accuracy in classification performance when identifying movements performed by novel participants can be attributed in part to the high degree of variability in movement performance and joint angles achieved between participants, exemplified in Figure 4. Grouping similar movement classes increases the range of potential comparators within the feature space by collapsing the movement classes based on the reported *FN*s. It is possible, however, that improved class separation between these novel movement classes could be improved by tuning the parameters in Equation (8).

## 4. Discussion

In this study, a mDTW-distance-based *k*NN algorithm for the recognition of motions frequently adopted in occupational settings based on inertial sensor data was developed. Joint angles of the left and right ankles, knees, and hips were segmented into motion templates to populate the classification feature space, and subsequently, these templates—representing 12 movements frequently performed in occupational settings—were compared, using DTW, to continuous movement sequences. The weighted sum of these DTW-based distances between waveforms was then calculated and a nearest neighbour to the unknown movement sequence was determined based on the minimum mDTW distance, calculated between all templates.

The classification framework presented in this study utilizes previously estimated joint angles rather than raw IMU data to ensure an interpretable association between the classification inputs and the output motion classes. While many machine learning-based approaches can be applied to the classification of human motion, several of these utilize deep learning to map connections between inputs and outputs through what is colloquially referred to as a “black box” [44]. Despite the demonstrated success of these approaches across a broad array of applications [45,46,47,48,49], recent efforts in the development of explainable or interpretable machine learning have sought to create “white box” algorithms, from which the link between inputs and results are transparent, interpretable, and explainable [44,50,51]. Given the evaluation of high-knee-flexion postures adopted in occupational settings goes beyond simply identifying their occurrence, it was important to develop a classification framework based on kinematic values which could be easily understood, interpreted, and further analyzed by users within both academic and occupational communities. The use of a computationally simple *k*NN classification model based on mDTW distances therefore satisfies these objectives and will serve to advance the research and knowledge surrounding exposures of high-knee flexion beyond laboratory-based settings.

### 4.1. Model Accuracies

To our knowledge this paper constitutes the first study to apply the proposed mDTW-distance-based classification technique to the identification of high-knee-flexion movements. The accuracy of the developed model to classify movement sequences based on predefined templates was evaluated both on participants for whom a portion of their data were and were not included in the labelled classification feature space. The highest accuracies were achieved when classifying the testing data, where a portion of a given participant’s data had been included in this feature space. In practice, these classification accuracies could be achieved by performing a brief collection of supervised movement trials from novel participants, which could be added to the algorithm building dataset prior to the measurement of unsupervised exposure data in workplace settings. Experimental results demonstrate that the model classified eleven of the twelve occupational motions in novel participant data with 55.6% accuracy when classified independently, or, when grouping motions based on their similarities, with an accuracy of 75.7%. The developed model was not capable of classifying sequences of walking data based on a single step template in its current implementation. Combining the models created for classifying all motions independently (tested on novel participant data) and for classifying grouped motions may provide a means by which higher accuracies in classification can be achieved for generalized movement identification while providing postural granularity at a lower level of certainty when classifying novel participant data. This solution may be of use in cases when the identification of specific movements is not the primary goal.

Based on the balanced accuracies calculated for each model, as presented in Table 4, the discrepancies in motion class representation within the model feature space (resulting from the training dataset) represent no significant impact on classification accuracy overall nor on the discrimination between movement classes. It is noted that, given the number of samples included in the feature space, balanced accuracies, in general, increase in relation to the predicted accuracies. This is due to the inclusion of sensitivity and specificity in the calculation, resulting in score inflation for poorly classified movements due to the high ratio of *TN*s to *FP*s in these multi-class classification models.

### 4.2. Model Sensitivities

Classification sensitivities of all high-flexion postures using the tuned and tested classification model were found to be well above the rate of chance (7.7%). The decrease in motion class sensitivity between the testing dataset (the 20% of segmented movements withheld from the model building dataset, results presented in Table 2C) and the validation dataset (segmented movements from the 15 novel participants, results presented in Table 2D) may be attributable to overfitting of the model to the training data. It is possible that the model parameter tuning resulted in prediction abilities which were not generalizable to novel participants. Future work should seek to address this potential overfitting by exploring novel parameter tuning methods which would increase generalizability. Further, differences in model sensitivities could be attributable to inter-participant variability in joint ranges of motion and movement patterns leading to confusion in the classification of kinematically similar motions.

### 4.3. Model Specificities

Specificity results appear to be the least affected by the model refinement, testing, and validation. This is likely due to the high number of movement classes included in the classification models. The ratio of *TN*s for any given movement in comparison with the incorrectly identified *FP*s is probabilistically high when classifying multiple movements, however, it does indicate the ability of the mDTW-distance-based *k*NN to distinguish between movement classes instead of favouring one particular class. Consistently, when testing and validating the tuned parameter model, the lowest specificities were observed in stooping, a result of the high rates of *FP* misclassifications of flatfoot and heels-up squatting motions within the stooping output class.

### 4.4. Misclassifications

The highest rates of misclassifications in the tuned parameter model testing and validation were observed in the FS and HS motion classes, where movement sequences were frequently misidentified as belonging to the opposite squatting motion or as stooping. We note that in model testing using movement sequences, a greater proportion of squatting sequences were misclassified as STP than in model validation using novel participant movement sequences; for FS, misclassifications were roughly evenly divided between HS and STP, while for HS, misclassification rates as FS were nearly double those as STP. There are several likely reasons for these misclassifications which will here be explored. Firstly, model confusion may be attributable to ergonomic-based strategies adopted during stooping given that many had received advanced training in safe lifting strategies. Therefore, when prompted to stoop as though they were lifting a child, their motions were characterized by a greater degree of flexion about the knees than would be expected in a typical stoop (characterized by isolated flexion about the hips), and as such would more closely resemble a stiff FS. Additionally, achievable depth in squatting is determined by multiple factors including ankle and foot mobility, foot positioning, and torso inclination. Participants with low degrees of ankle mobility were observed to have greater difficulty achieving high-flexion angles at the knees in FS while participants with low degrees of foot mobility were observed to have difficulty maintaining balance and achieving high-knee-flexion angles in HS. The positioning of the feet was also observed to impact the achievable degree of knee flexion in both FS and HS as a wider stance was observed to enable external rotation at the hips, thereby allowing most individuals to achieve greater squat depth. However, given that participants took a step forward prior to squatting, many participants squatted with their feet between hip and shoulder distance apart rather than adopting a wider stance. Finally, increased torso inclination (resulting in increased hip flexion) was observed as a strategy adopted by many participants to increase balance while squatting despite instructions to maintain alignment between the shoulders and feet. All of these factors affect the joint kinematics of the ankles, knees, and hips and have likely contributed to the misclassifications between the squatting and kneeling postures.

Similar factors influence the misclassifications observed between the other movement classes included in this study. Thigh and calf circumference as well as flexibility contribute to participants’ ability to achieve high-knee-flexion angles in DK and PK movements. Participants unable to achieve high-flexion angles at the knee often also exhibited greater hip flexion, and given that foot placement was not prescribed for the SK motions, these postural adaptations likely resulted in misclassifications between the supported and unsupported kneeling motions. Further, for DK specifically, these adaptations resulted in similar kinematics to those adopted in both the CCS and ACS motions. Misclassifications between the DK and PK motions are likely attributable to the low weightings assigned to ankle angles in Equation (8), while misclassifications between SS or CLS and both DK and PK likely result from the lack of a term representing hip internal/external rotation in Equation (8), and this constitutes the primary kinematic difference between these postures. Finally, misclassification rates between CCS and ACS are likely attributable to participant height differences leading to differences in joint angles within the feature space from which comparisons were calculated. Future work might seek to include additional kinematic or anthropometric features within the distance metric in order to account for these classification errors and increase the separation between movement classes.

### 4.5. Limitations

While the proposed algorithm was capable of classifying high-flexion motions in the dataset here presented, there are limitations to this classifier which must be considered. The current classification model was developed using simulated occupational data. Despite encouraging participants to perform each movement naturally, the duration and approach into and out of each motion were controlled which likely affected the way participants moved. Moving forward, the classifier should be provided with a series of templates based on more realistic unconstrained movements.

Similarly, it must be noted that this dataset was composed of movements recorded in isolation, such that no sequence contained transitions from one motion class to another. While the data included in the model sufficiently captured both the descent and ascent phases of each motion, in occupational settings it is likely that individuals might transition between motions (i.e., transition from kneeling to sitting, or descend into a flatfoot squat but ascend through a heels-up squat). The developed algorithm would likely be unable to classify such postural transitions. Therefore, future work should explore the possibility of extending the classification ability of the model to distinguish between different phases of motion, ultimately enabling the classifier to successfully detect consecutive phases of different motion. Additional data would be required, however, for the classifier to identify transitional movements. One must additionally be aware that the linear weightings used here in the mDTW approach might not be robust when classifying transitions in continuous datasets. Given the subtle differences between many of the high-knee-flexion movements simulated in the current study, it might be appropriate to explore a non-linear approach to best distinguish between postures.

Finally, it is noted that while DTW has proven to be a valuable tool for the analysis and comparison of time series data with temporal variability, its application for classification and subsequent identification faces notable limitations when presented with signals of significantly different durations or amplitudes. The flexibility of non-linearly warping the time of signals may result in the loss of discriminative features when prioritizing alignment, which is particularly detrimental to applications of classification. Further, when signal segmentation is not performed to isolate a particular movement, the inclusion of other functionally distinct biological signals can lead to erroneous classifications given the inherent assumption of alignment between sequences on which DTW relies [33,52]. Similarly, when signals are of significantly different scales, as might be possible between the joint angles of two participants, these scale differences may result in misalignments between waveforms [52]. In the current paper, we attempted to minimize these classification errors by segmenting signals based on movement performed and linearly interpolating each movement to 101 data points and scale normalizing between −1 and 1. In free-living data, however, it is likely that the duration of movements, the scale of these continuous signals, as well as the sequential nature of their occurrences would affect the predictive ability of the model. As such, future development should investigate the effects of these signal variations on classification outcomes.

## 5. Conclusions

In this study, successful classification of eleven movements was achieved using the proposed mDTW-distance-based Nearest Neighbour classification algorithm. A combination of weighted DTW distances calculated between motion templates and continuous movement sequences representing joint angles for the ankles, knees, and hips was found to be effective for the classification of these movements frequently performed occupationally. The generally high classification rates achieved when classifying data from individuals both included and precluded from the algorithm training–testing dataset indicate strong potential for the proposed model’s application to the quantitative measurement of postural requirements in occupational settings. Future work should seek to include additional kinematic indicators which might aid in the separability of motion classes, investigate means of compensating for signal variations in time and magnitude, and expand this classifier to identify separate phases of motion to successfully identify high-flexion postures in real-world settings.

## Figures and Tables

**Table 1:** Description of the thirteen simulated motions commonly adopted in occupational settings. Each motion analyzed has been illustrated in Figure 1.

Motions	Description
Heels-Up Squatting (HS)	Forefeet are in contact with the ground, at hip width or greater distance apart, while the heels are raised. The knees are anterior to the feet while the buttocks rest as close to the heels as possible, with the chest raised such that the shoulders are roughly superior to the feet.
Flatfoot Squatting (FS)	Feet are flat on the ground, at hip width or greater distance apart. The knees are driving toward the shoulders, located superiorly yet in line with the feet, while the tailbone is typically pointed to the ground.
Dorsiflexed Kneeling (DK)	Symmetrical kneeling, with flexed forefeet, so that the inferior aspect of the head of the metatarsals and the plantar aspect of the toes are in contact with the ground. The buttocks rest as close to the heels as possible and the torso is perpendicular with the ground.
Plantarflexed Kneeling (PK)	Symmetrical kneeling, where the superior aspect of the foot is in contact with the ground, the buttocks rest as close to the heels as possible, and the torso is perpendicular with the ground.
Single Arm Supported Kneeling (SAK)	Both knees and the dominant hand are in contact with the ground, roughly inferior to the hips and shoulder, respectively. Body weight is evenly distributed between all three contact points. Foot position was not controlled in this posture.
Double Arm Supported Kneeling (DAK)	Similar to SAK with both hands in contact with the ground, so that the body weight is evenly distributed between the four contact points. Again, foot position was not controlled in this posture.
Sitting on an Adult-Sized Chair (ACS)	Buttocks are seated on an adult-sized stool wherein the seat pan height is roughly at knee level. Both feet are planted on the ground inferior to the knees.
Sitting on a Child-Sized Chair (CCS)	Buttocks are seated on a child-sized chair wherein the seat pan height is below knee level. Both feet are planted on the ground inferior to the knees.
Crossed Leg Sitting (CLS)	Buttocks are seated on the ground. Legs are bent so that the feet are crossed in front of the body. Participants were permitted to use their hands when descending into and rising from the posture as needed.
Side Sitting (SS)	Similar to a kneeling posture, however, the buttocks have moved laterally from the heels so that one hip rests on the ground while hands rest on the superior thigh. This posture was performed to both the left and right sides.
Side Leaning (SL)	Similar to the side sitting posture, with a single hand in contact with the ground, roughly inferior to the shoulder, for additional support.
Stooping (STP)	Movement primarily involving a hinge about the hips. The knee flexion angle typically does not exceed 90°. Participants were instructed to perform this task as if they were reaching to lift a child or object from an estimated height of 0.5 m above the floor.
Standing (STD)	Participants were asked to stand with arms crossed across the chest and lower limbs stacked such that the ankles and knees were roughly inferior to the hips.

**Table 2:** Sensitivity-based confusion matrices for the classification of twelve occupational-inspired motions based on (**A**) the five-fold cross-validation of the algorithm training templates, (**B**) the testing of segmented data using a model based on tuned k and the mDTW weighting parameters, (**C**) the testing of movement sequences using the tuned parameter model, and (**D**) the validation of the tuned parameter model on novel participant movement sequences. All values are expressed as percentages relative to the total number of classifications for each occupational-inspired motion. Bolded cells denote correct classifications. The twelve motions analyzed have been illustrated in Figure 1. * Target Class represents the correct class of each template or sequence. ** Output Class represents the class predicted by the classifier.

**A**													
Five-Fold Cross-Validated *k*NN on Training Data													
Target Class *	DK	**71.8**	10.3	5.1	5.1	2.6	0.0	5.1	0.0	0.0	0.0	0.0	0.0
	PK	0.0	**100**	0.0	0.0	0.0	0.0	0.0	0.0	0.0	0.0	0.0	0.0
	FS	0.0	0.0	**81.6**	10.5	5.3	2.6	0.0	0.0	0.0	0.0	0.0	0.0
	HS	0.0	0.0	27.0	**64.9**	5.4	2.7	0.0	0.0	0.0	0.0	0.0	0.0
	CCS	2.6	0.0	0.0	0.0	**68.4**	26.3	0.0	0.0	0.0	0.0	2.6	0.0
	ACS	0.0	0.0	0.0	2.6	5.3	**92.1**	0.0	0.0	0.0	0.0	0.0	0.0
	SK	7.3	2.4	2.4	0.0	2.4	0.0	**82.9**	0.0	0.0	0.0	2.4	0.0
	STP	4.8	0.0	4.8	19.0	0.0	0.0	0.0	**71.4**	0.0	0.0	0.0	0.0
	STD	0.0	0.0	0.0	5.6	0.0	5.6	0.0	11.1	**77.8**	0.0	0.0	0.0
	CLS	3.4	3.4	0.0	3.4	0.0	0.0	3.4	0.0	0.0	**72.4**	13.8	0.0
	SS	1.7	0.8	0.0	0.0	1.7	0.8	1.7	0.0	0.0	5.0	**88.2**	0.0
	WLK	0.0	0.0	0.0	0.0	0.0	0.0	0.0	0.0	0.0	0.0	0.0	**100**
		DK	PK	FS	HS	CCS	ACS	SK	STP	STD	CLS	SS	WLK
Output Class **													
**B**													
Tuned Parameter *k*NN on Segmented Testing Data													
Target Class	DK	**76.9**	5.1	5.1	2.6	5.1	0.0	5.1	0.0	0.0	0.0	0.0	0.0
	PK	0.0	**100**	0.0	0.0	0.0	0.0	0.0	0.0	0.0	0.0	0.0	0.0
	FS	0.0	0.0	**81.6**	13.2	2.6	2.6	0.0	0.0	0.0	0.0	0.0	0.0
	HS	0.0	0.0	24.3	**73.0**	2.7	0.0	0.0	0.0	0.0	0.0	0.0	0.0
	CCS	2.6	0.0	0.0	0.0	**68.4**	26.3	0.0	0.0	0.0	0.0	2.6	0.0
	ACS	0.0	2.6	0.0	2.6	2.6	**92.1**	0.0	0.0	0.0	0.0	0.0	0.0
	SK	7.3	0.0	2.4	0.0	2.4	0.0	**85.4**	0.0	0.0	0.0	2.4	0.0
	STP	4.8	0.0	9.5	9.5	0.0	0.0	0.0	**76.2**	0.0	0.0	0.0	0.0
	STD	0.0	0.0	0.0	0.0	5.6	0.0	0.0	5.6	**61.1**	5.6	22.2	0.0
	CLS	0.0	3.4	0.0	3.4	0.0	0.0	3.4	0.0	0.0	**75.9**	13.8	0.0
	SS	1.7	0.8	0.0	0.0	0.0	0.0	0.0	0.0	0.0	5.9	**91.6**	0.0
	WLK	0.0	0.0	0.0	0.0	0.0	0.0	0.0	0.0	0.0	0.0	0.0	**100**
		DK	PK	FS	HS	CCS	ACS	SK	STP	STD	CLS	SS	WLK
Output Class													
**C**													
Tuned Parameter *k*NN on Testing Movement Sequences													
Target Class	DK	**100**	0.0	0.0	0.0	0.0	0.0	0.0	0.0	0.0	0.0	0.0	0.0
	PK	0.0	**87.2**	0.0	2.6	2.6	0.0	0.0	2.6	2.6	0.0	2.6	0.0
	FS	2.6	0.0	**57.9**	7.9	0.0	0.0	2.6	28.9	0.0	0.0	0.0	0.0
	HS	2.6	0.0	18.4	**52.6**	0.0	0.0	0.0	26.3	0.0	0.0	0.0	0.0
	CCS	2.7	0.0	0.0	2.7	**89.2**	5.4	0.0	0.0	0.0	0.0	0.0	0.0
	ACS	0.0	0.0	0.0	2.6	10.5	**84.2**	0.0	2.6	0.0	0.0	0.0	0.0
	SK	2.4	2.4	0.0	0.0	0.0	0.0	**95.1**	0.0	0.0	0.0	0.0	0.0
	STP	4.8	0.0	0.0	4.8	0.0	0.0	0.0	**90.5**	0.0	0.0	0.0	0.0
	STD	0.0	0.0	0.0	0.0	0.0	0.0	0.0	0.0	**89.5**	0.0	10.5	0.0
	CLS	0.0	0.0	0.0	0.0	0.0	0.0	0.0	0.0	0.0	**96.6**	3.4	0.0
	SS	0.8	0.8	0.0	0.0	0.8	2.5	0.0	0.0	0.0	2.5	**92.4**	0.0
	WLK	14.3	0.0	0.0	0.0	0.0	4.8	4.8	47.6	14.3	0.0	14.3	**0.0**
		DK	PK	FS	HS	CCS	ACS	SK	STP	STD	CLS	SS	WLK
Output Class													
**D**													
Tuned Parameter *k*NN on Novel Participant Validation Movement Sequences													
Target Class	DK	**63.2**	6.9	0.0	1.1	9.2	9.2	4.6	0.0	0.0	0.0	5.7	0.0
	PK	1.1	**79.3**	0.0	0.0	0.0	0.0	8.0	0.0	0.0	0.0	11.5	0.0
	FS	6.9	1.1	**35.6**	25.3	2.3	1.1	0.0	27.6	0.0	0.0	0.0	0.0
	HS	3.4	2.3	32.2	**39.1**	4.6	0.0	0.0	18.4	0.0	0.0	0.0	0.0
	CCS	0.0	0.0	1.2	6.0	**67.9**	22.6	1.2	1.2	0.0	0.0	0.0	0.0
	ACS	0.0	0.0	1.2	1.2	30.5	**65.9**	0.0	0.0	0.0	0.0	1.2	0.0
	SK	20.0	9.4	0.0	1.2	4.7	3.5	**48.2**	0.0	0.0	0.0	12.9	0.0
	STP	0.0	0.0	9.3	4.7	0.0	4.7	4.7	**76.7**	0.0	0.0	0.0	0.0
	STD	0.0	0.0	1.2	3.5	2.4	2.4	1.2	4.7	**76.5**	0.0	4.7	3.5
	CLS	1.3	4.3	3.9	0.0	3.9	1.3	0.0	0.0	0.0	**61.8**	26.3	0.0
	SS	5.6	7.3	3.1	3.5	5.9	4.2	3.8	0.3	0.0	12.6	**53.5**	0.0
	WLK	0.0	0.0	0.0	0.0	6.7	4.4	2.2	75.6	2.2	0.0	8.9	**0.0**
		DK	PK	FS	HS	CCS	ACS	SK	STP	STD	CLS	SS	WLK
Output Class													

**Table 3:** Specificity values achieved in the classification of twelve occupational-inspired motions. All values are expressed as percentages relative to the total number of classified waveforms belonging to other motions. The twelve motions analyzed have been illustrated in Figure 1.

Motion	DK	PK	FS	HS	CCS	ACS	SK	STP	STD	CLS	SS	WLK
Classifier Type												
Cross-Validated *k*NN	97.8	98.1	96.3	96.6	97.3	96.2	98.6	99.5	100	98.4	98	100
Tuned Parameter *k*NN on Segmented Data	98.1	98.6	96.4	97.4	98.2	97.1	99.2	99.7	100	97.9	97.0	99.7
Tuned Parameter *k*NN on Movement Sequences	97.6	99.5	98.2	98.2	98.4	98.4	99.5	92.2	98.9	99.2	97.1	100
Novel Participant Movement Sequences	93.0	93.6	92.8	93.1	89.5	92.1	95.7	88.3	99.8	94.3	89.8	99.5

**Table 4:** Balanced accuracy values achieved in the classification of twelve occupational-inspired motions. All values are expressed as percentages. The twelve motions analyzed have been illustrated in Figure 1.

Motion	DK	PK	FS	HS	CCS	ACS	SK	STP	STD	CLS	SS	WLK
Classifier Type												
Cross-Validated *k*NN	84.7	99.0	88.9	80.7	82.9	94.2	90.8	85.5	88.9	85.4	93.1	100
Tuned Parameter *k*NN on Segmented Data	87.5	99.3	89.0	85.2	83.3	94.6	92.3	88.0	80.6	86.9	94.3	99.9
Tuned Parameter *k*NN on Movement Sequences	98.8	93.3	78.1	75.4	93.8	91.3	97.3	91.3	94.2	97.9	94.8	50.0
Novel Participant Movement Sequences	78.1	86.5	64.2	66.1	78.7	79.0	92.0	82.5	88.1	78.1	71.7	49.8

**Table 5:** Sensitivity, specificity, and balanced accuracy values achieved in the classification of occupational-inspired motions when grouped based on similarity of motions such that kneeling encompasses dorsiflexed, plantarflexed, and supported kneeling; squatting encompasses flatfoot and heels-up squatting; chair sitting encompasses sitting on adult- and child-sized chairs; and floor sitting encompasses crossed leg and side sitting. All values are expressed as percentages.

Motion	Kneeling	Squatting	Chair Sitting	Stooping	Standing	Floor Sitting	WLK
Performance Measure							
Sensitivity	80.3	66.1	93.4	76.7	76.5	70.7	0.0
Specificity	81.3	86.2	81.7	88.3	99.8	82.8	99.5
Balanced Accuracy	81.0	76.0	88.0	83.0	88.0	77.0	49.8

## Data Availability

The data presented in this study are available on request from the corresponding author due to ethical restrictions.
